# Global Transcriptomic Analysis of the Candida albicans Response to Treatment with a Novel Inhibitor of Filamentation

**DOI:** 10.1128/mSphere.00620-19

**Published:** 2019-09-11

**Authors:** Jesus A. Romo, Hao Zhang, Hong Cai, David Kadosh, Julia R. Koehler, Stephen P. Saville, Yufeng Wang, Jose L. Lopez-Ribot

**Affiliations:** a Department of Biology, The University of Texas at San Antonio, San Antonio, Texas, USA; b South Texas Center for Emerging Infectious Diseases, The University of Texas at San Antonio, San Antonio, Texas, USA; c Department of Microbiology, Immunology and Molecular Genetics, University of Texas Health Science Center at San Antonio, San Antonio, Texas, USA; d Division of Infectious Diseases, Boston Children’s Hospital/Harvard Medical School, Boston, Massachusetts, USA; Carnegie Mellon University

**Keywords:** *Candida albicans*, candidiasis, filamentation, antivirulence

## Abstract

The opportunistic pathogenic fungus Candida albicans can cause devastating infections in immunocompromised patients. Its ability to undergo a morphogenetic transition from yeast to filamentous forms allows it to penetrate tissues and damage tissues, and the expression of genes associated with a number of pathogenetic mechanisms is also coordinately regulated with the yeast-to-hypha conversion. Therefore, it is widely considered that filamentation represents one of the main virulence factors of C. albicans. We have previously identified *N*-[3-(allyloxy)-phenyl]-4-methoxybenzamide (compound 9029936) as the lead compound in a series of small-molecule inhibitors of C. albicans filamentation and characterized its activity both *in vitro* and *in vivo*. This compound appears to be a promising candidate for the development of alternative antivirulence strategies for the treatment of C. albicans infections. In this study, we performed RNA sequencing analysis of samples obtained from C. albicans cells grown under filament-inducing conditions in the presence or absence of this compound. Overall, treatment with compound 9029936 resulted in 618 upregulated and 702 downregulated genes. Not surprisingly, some of the most downregulated genes included well-characterized genes associated with filamentation and virulence such as *SAP5*, *ECE1* (candidalysin), and *ALS3*, as well as genes that impact metal chelation and utilization. Gene ontology analysis revealed an overrepresentation of cell adhesion, iron transport, filamentation, biofilm formation, and pathogenesis processes among the genes downregulated during treatment with this leading compound. Interestingly, the top upregulated genes suggested an enhancement of vesicular transport pathways, particularly those involving SNARE interactions.

**IMPORTANCE** These results from whole-genome transcriptional profiling provide further insights into the biological activity and mode of action of a small-molecule inhibitor of C. albicans filamentation. This information will assist in the development of novel antivirulence strategies against C. albicans infections.

## Abstract (precis)

These results from whole-genome transcriptional profiling provide further insights into the biological activity and mode of action of a small-molecule inhibitor of C. albicans filamentation. This information will assist in the development of novel antivirulence strategies against C. albicans infections.

## INTRODUCTION

The opportunistic pathogenic fungus Candida albicans is a common member of the human microbiota (1, 2). However, this otherwise normal commensal of humans is also capable of causing a range of diseases in immunocompromised and medically compromised individuals, as well as those on heavy doses of antibiotics. Candidiasis carries mortality rates of 40 to 60% (3) and current therapeutic options are few, mostly restricted to polyenes, azoles, and echinocandins. Use of these antifungals is limited by toxicity, drug-drug interactions, and the emergence of resistance (4–6). Clearly, novel approaches to antifungal drug development are urgently needed (7, 8).

C. albicans is able to undergo reversible morphogenetic conversions between yeast and filamentous morphologies, which are intimately linked to the pathogenesis of candidiasis, as filaments can invade tissues and cause damage (9–12), and filamentation also plays a central role in biofilm formation (13–18). Moreover, a number of other pathogenetic properties of C. albicans, such as adhesive interactions and production of proteolytic enzymes and toxins, are also coordinately regulated with the morphological conversion to hyphae (1, 19–26). Altogether, it is now widely accepted that filamentation constitutes one of the main virulence factors associated with the pathogenesis of C. albicans, thereby representing an attractive, yet unexploited, target for the development of a novel antivirulence strategy for the treatment of candidiasis (7, 27–29). We have previously reported on the *in vitro* and *in vivo* activities of compound 9029936 (30, 31). This compound, with a biaryl amide core structure, was originally identified as one of the top hits in a screen of 30,000 small-molecule compounds from a commercially available chemical library in a search for inhibitors of C. albicans filamentation (30, 31). Interestingly, compound 9029936 inhibits filamentation of all C. albicans strains tested (including collection strains and clinical isolates) and in all different media used to induce the yeast-to-hypha transition, indicating that it probably impacts a common node of the multiple signaling pathways that control hypha formation under different environmental conditions (30, 31). It displays a good safety profile and potent *in vivo* activity in different animal models of C. albicans infections, and therefore, it represents a promising candidate for the development of novel antifungal approaches targeting virulence instead of cell proliferation or viability (as current fungistatic or fungicidal drugs do). In this study, we used RNA sequencing (RNA-seq) to investigate the impact of treatment with compound 9029936 on the whole transcriptome of C. albicans. Our results indicate that exposure of cells to the compound leads to a downregulation of genes and pathways associated with C. albicans virulence, providing further insights into the antivirulence mode of action of this small-molecule compound.

## RESULTS AND DISCUSSION

### Global transcriptomic changes of C. albicans in response to treatment with compound 9029936.

**FIG 1:**
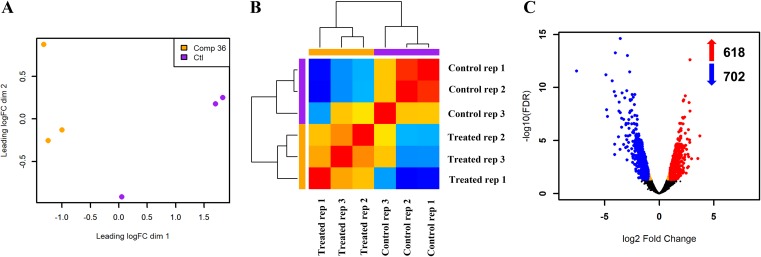
Overall transcriptomic changes of C. albicans growing under filament-inducing conditions in response to treatment with compound 9029936. (A) Principal-component analysis (PCA) plot showing the level of correlation and reproducibility among control untreated samples (purple) and samples treated with 5 μM compound 9029936 (orange). (B) Hierarchical-clustering heat map of gene expression data. The color scale indicates the degree of correlation (red, high correlation; blue, low correlation), while the height of the dendrogram branches represents the variability in gene expression between samples. (C) Volcano plot showing the significantly upregulated (red) and downregulated (blue) genes in samples treated with compound 9029936. A cutoff absolute value of log fold change >1 (2-fold change) was used. Adjusted *P* value < 0.05.

We investigated changes in C. albicans gene expression under filament-inducing conditions as a result of treatment with compound 9029936 through the use of RNA sequencing. To that end, C. albicans SC5314 was grown under strong filament-inducing conditions (yeast extract-peptone-dextrose [YPD] containing 10% fetal bovine serum [FBS] at 37°C) in the presence or absence of compound 9029936 at 5 μM. Cells were harvested after 90 min, since prior experiments have indicated that compound 9029936 exerts its inhibitory effects during the early stages of the morphological transition (30). The efficacy of treatment with this leading compound was monitored by microscopic examination, which corroborated the fact that compound 9029936 was able to block filamentation, while the untreated samples filamented normally (data not shown). Experiments were conducted in triplicate and RNA was extracted from the different cultures. Samples were then subjected to RNA sequencing using an Illumina HiSeq 2000 platform (as described in Materials and Methods). Table S1 in the supplemental material shows the sequence reads produced from all samples, with an average of 38,090,680 reads produced per sample and with 90% or more successfully mapped to the C. albicans strain SC5314 reference genome (assembly 21, downloaded from the *Candida* Genome Database [http://www.candidagenome.org/]). Principal-component analysis (PCA) and hierarchical clustering were applied to provide a visual representation of the transcriptomic similarities between samples treated with compound 9029936 and the untreated controls. Samples from different conditions (presence or absence of compound 9029936) clustered separately, while those from the same conditions clustered together, indicating a high level of correlation among samples, as well as distinctive transcriptome profiles (Fig. 1A and B). Analyses of the RNA sequencing data clearly indicate that compound 9029936 has a profound effect on C. albicans gene expression leading to vast alterations in the transcriptome. For this analysis, genes that showed greater than a 2-fold (up or down) change in their level of expression were considered differentially expressed, and the cutoff for statistical significance used a Benjamini-Hochberg adjusted *P* value of <0.05. A total of 1,320 genes showed a significant difference in expression between samples treated with 5 μM compound 9029936 and those left untreated under filament-inducing conditions (Fig. 1C). Among these differentially expressed genes (DEGs), 618 were upregulated and 702 were downregulated in the compound-treated samples (relative to the untreated controls).

10.1128/mSphere.00620-19.2 TABLE S1 Sequenced reads obtained from the sample set from RNA sequencing experiments. Download Table S1, PDF file, 0.2 MB. Copyright © 2019 Romo et al. 2019 Romo et al. This content is distributed under the terms of the Creative Commons Attribution 4.0 International license.

### Treatment with compound 9029936 results in downregulation of genes associated with C. albicans pathogenetic processes.

**FIG 2:**
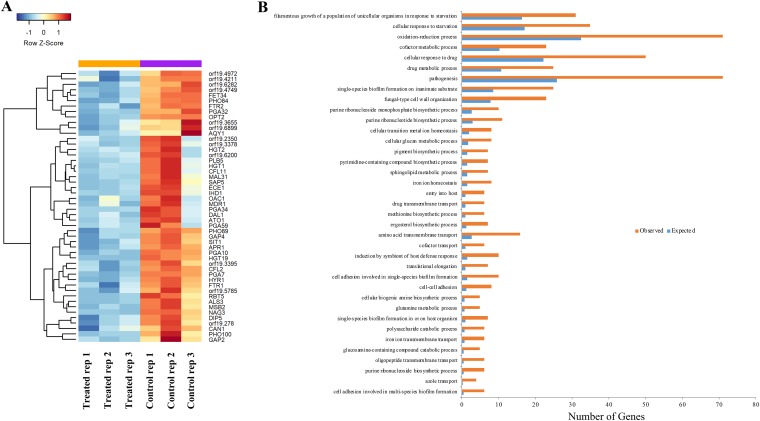
The top 50 downregulated genes and overrepresented GO terms for biological processes downregulated in response to treatment with compound 9029936 under filament-inducing conditions. (A) Heat map displaying the top 50 downregulated genes in C. albicans cells treated with 5 μM compound 9029936 versus untreated control cultures. (B) Bar graph representation of significantly overrepresented GO terms for biological processes with downregulated genes in RNA sequencing analysis in response to treatment with 5 μM compound 9029936.

We first focused our attention on those genes whose expression was downregulated during treatment with our leading compound. Figure 2A shows a heat map of the top 50 downregulated genes. This list of most downregulated genes includes some uncharacterized open reading frames (ORFs), such as *orf19*.4749 and *orf19*.6282, whereas *orf19*.4972 (*OFI1*) encodes a putative transcription factor that has been shown to be involved in the regulation of white-opaque switching and filamentous growth (32, 33). Two genes (*FET31* and *FET34*), encoding the major multicopper oxidases (MCOs) that form part of the high-affinity iron uptake system, are normally activated in response to iron-limited conditions inside the host and have been shown to play important roles in hyphal development and virulence (34, 35). Interestingly, *FTR2* and, to a lesser extent, *FTR1*, encoding high-affinity iron permeases with essential roles in virulence (34, 36–38), were also in this list of most-downregulated genes, as was *RBT5* (*PGA1*), encoding a glycosylphosphatidylinositol (GPI)-anchored cell wall protein involved in the utilization of hemin and hemoglobin for iron in the host (36, 39–41).

Besides iron uptake, both morphogenetic conversions and invasion of tissues by C. albicans require phosphate transporters (42), and of note, the eighth most downregulated gene was *PHO84*, which encodes a high-affinity phosphate transporter that intersects with the TOR pathway and was recently suggested as a potential antifungal target (43, 44). *PHO84* and two other *PHO* genes (*PHO89* and *PHO100*) in the 50 most significantly downregulated genes are induced during phosphate starvation (45), indicating that treatment with compound 9029936 leads to downregulation of genes associated with phosphate acquisition with important roles in oxidative stress and virulence.

**FIG 3:**
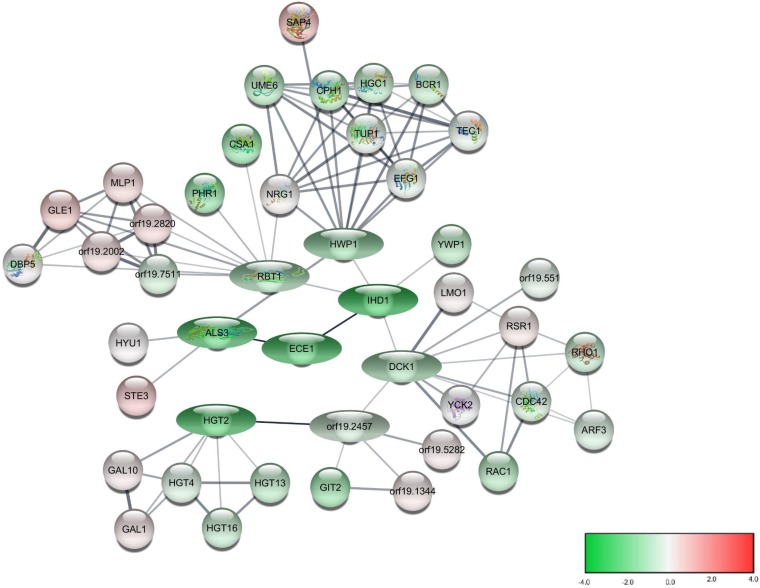
Network visualization for predicted protein-protein interactions and expression levels of genes involved in the core filamentation response network. The STRING database is a curated knowledge database of known and predicted protein-protein associations. The genes whose protein products are known or predicted to have direct protein-protein interaction are highlighted. The eight genes identified as forming part of the C. albicans core filamentation response network are represented by ovals. All other interacting genes are represented by circles. The relative expression levels of genes in C. albicans cells treated with 5 μM compound 9029936 versus untreated control cultures are represented by a gradient of colors from green (downregulation) to red (upregulation) indicating log2 fold changes. The input genes are drawn as filled nodes when their protein structures are known or predicted, while unfilled nodes are used for the additional genes with unknown protein structures.

As expected from a filamentation inhibitor, featured prominently in this group of top 50 genes downregulated during treatment with compound 9029936 are well-characterized genes associated with filamentation and pathogenetic mechanisms of C. albicans (i.e., adhesion and production of proteolytic enzymes and toxins), including *SAP5*, and a majority of genes that form part of the C. albicans core filamentation response network (46), such as *ECE1*, *ALS3*, *IHD1*, and *HGT2*. Sap5 is a well-known secreted aspartyl proteinase important for virulence and pathogenesis during filamentation and biofilm formation by C. albicans (47–50). Ece1p, long associated with C. albicans hypha formation, with its internal small peptide candidalysin, is a vital virulence factor via which C. albicans permeabilizes host epithelial membranes, enabling tissue invasion (21). Als3 (a member of the agglutinin-like sequence adhesins) is utilized by C. albicans during filamentation for epithelial and endothelial adhesion, and null mutants are defective in these adhesive properties (51). Furthermore, Als3p is also known to play a role in iron acquisition, which, as mentioned previously, is crucial for fungal pathogenesis (19). Interestingly, although not in the top 50 list, levels of expression of all other members of the *ALS* gene family were significantly downregulated too. *IHD1* encodes a relatively poorly characterized GPI-anchored protein induced during hypha formation (52, 53). Little information is available for *HGT2*, except that it constitutes part of the core filamentation response network in C. albicans (46); it is homologous to the high-affinity glucose transporter *HGT1*, which also plays multiple roles in virulence and in the evasion of immune defenses (54) and, interestingly, was also among the top 50 genes most downregulated by treatment with our compound. Also in this list of top 50 downregulated genes were *PLB5*, encoding a phospholipase with a role in virulence (55), and *HYR1*, encoding a prototypical hypha-specific cell wall protein (56). Other genes encoding surface adhesins, such as *PGA32* (also induced in high iron), *PGA59*, and *MSB2*, were also downregulated, pointing to potential secondary effects on the cell surface as a consequence of the inhibition of hypha formation. Of note, although absent from the top 50 downregulated genes, expression of all other genes coding for the other members of the core filamentation response network was also significantly downregulated during treatment with compound 9029936. These included *HWP1*, encoding a hypha-specific adhesin complementary to Als3p (57), *RBT1*, encoding a hyphal adhesin related to Hwp1p involved in mating and filamentation (58), and *DCK1*, coding for a putative guanine nucleotide exchange factor required for embedded filamentous growth (59), as well as the uncharacterized *orf19*.2457. The data were also used to generate a protein interaction network using the STRING (Search Tool for the Retrieval of Interacting Genes) database; results are shown in Fig. 3. This figure depicts protein-protein associations for genes in the C. albicans core filamentation response, based on both known and predicted interactions of their corresponding protein products. The gradient of colors indicates differential levels of expression from RNA sequencing results for each different gene in cells treated with compound 9029936.

Importantly, the set of genes that is downregulated in response to treatment with compound 9029936 shows significant overlap with the set of genes previously shown to be strongly upregulated by C. albicans in response to serum and temperature (60), one of the strongest filament-inducing conditions. Many of these genes are involved in a diverse array of processes important for pathogenicity. These findings confirm our previous results (30, 31) suggesting that compound 9029936 may target an upstream regulator (i.e., transcription factor) that controls expression of the C. albicans filamentous growth program.

To identify larger patterns in differential gene expression and obtain overall insight into the impact of compound 9029936, Gene Ontology (GO) terms were assigned to all of the genes in the C. albicans genome and we then compared terms for the downregulated genes to a background of all terms. We found a total of 37 GO terms that were overrepresented (enriched) in this analysis (Fig. 2B). Somewhat unsurprisingly due to the antivirulence nature of the compound, filamentation, biofilm formation, cell adhesion, and pathogenesis were among the most enriched gene classes found to be downregulated. These results further support the finding that the morphogenetic transition is the main biological process affected by treatment with this compound. Other notable overrepresented GO terms for downregulated genes were those involved in response to starvation, oxidation reduction, and iron homeostasis and transport, all of which have been linked to C. albicans pathogenesis (Fig. 2B).

C. albicans displays a high degree of metabolic plasticity that greatly contributes to virulence by allowing it to rapidly adapt to different niches within the host where nutrient availability may be limited. This capacity to sense its surroundings and adapt to changing microenvironments in the human host is critical for both C. albicans survival as a commensal and as an opportunistic pathogen (61, 62). Moreover, morphological transitions in C. albicans are accompanied by changes in metabolism, giving each morphological state its own metabolic fingerprint (63–66). Thus, we also performed Kyoto Encyclopedia of Genes and Genomes (KEGG) analysis based on the sequencing data in order to detect which metabolic pathways were impacted by treatment with compound 9029936. This analysis detected a total of 20 KEGG pathways that were significantly downregulated by treatment with our leading compound under filament-inducing conditions (Table S2). These overwhelmingly included pathways involved in carbon metabolism, biosynthesis and metabolism of amino acids, and biosynthesis of secondary metabolites. Thus, it would seem that treatment with compound 9029936 leads to the downregulation of a multitude of metabolic and biosynthetic pathways associated not only with the morphogenetic yeast-to-hypha transition but also with the pathogenesis of candidiasis, since induction of these pathways is required for C. albicans to adapt to different environments and display its full virulence potential within the host (60, 67–74). Interestingly, we did not observe an overall downregulation of genes involved in endocytosis pathways, indicating that compound 9029936 functions differently from or has a mechanism of action different from those of some previously described inhibitors of C. albicans filamentation (75).

10.1128/mSphere.00620-19.3 TABLE S2 Significantly overrepresented KEGG pathways downregulated in C. albicans cells during treatment with compound 9029936. Download Table S2, XLSX file, 0.01 MB. Copyright © 2019 Romo et al. 2019 Romo et al. This content is distributed under the terms of the Creative Commons Attribution 4.0 International license.

### Treatment with compound 9029936 upregulates the expression of C. albicans genes required for vesicular transport.

**FIG 4:**
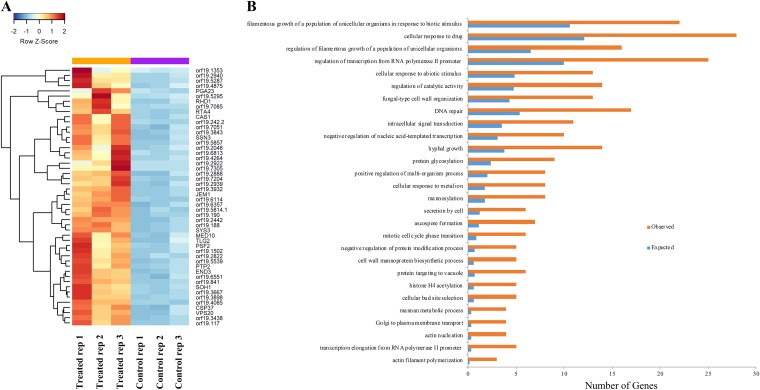
The top 50 upregulated genes and overrepresented GO terms for biological processes upregulated in response to treatment with compound 9029936 under filament-inducing conditions. (A) Heat map displaying the top 50 upregulated genes in C. albicans cells treated with 5 μM compound 9029936 versus untreated control cultures. (B) Bar graph representation of significantly overrepresented GO terms for biological processes with upregulated genes in RNA sequencing analysis in response to treatment with 5 μM compound 9029936.

In the analysis of the top 50 genes upregulated in response to treatment with compound 9029936 under filament-inducing conditions (Fig. 4A), a large number of uncharacterized genes (indicated by open reading frame numbers) with unknown functions emerged. In fact, of the top most upregulated genes, two are completely uncharacterized (*orf19*.1353 and *orf19*.5287), while one is predicted to play a role in mRNA splicing (*orf19*.4875) and one is predicted to be a putative v-SNARE of the endoplasmic reticulum (ER) membrane (*orf19*.2940/*BOS1*). Interestingly, several of the products encoded by upregulated genes are involved in vesicular transport within the cell. These included Bos1p (described above), Orf19.5875p, and Tlg1p/Tlg2p, all of which are predicted to encode syntaxin-like vacuolar t-SNAREs involved in vacuolar inheritance, and Orf19.2888p, which is predicted to play a role in protein complex assembly as well as early endosome-to-Golgi transport. Additionally, Orf19.5539p is also predicted to be involved in retrograde vesicle-mediated transport as well as Golgi-to-ER transport and SNARE complexes, whereas Gos1p is predicted to play a role in vesicle transport and fusion as well as SNARE complex localization. Orf19.841p is predicted to be involved in Golgi vesicle transport and Golgi membrane localization, while Sys3p plays a role in Golgi vesicle docking. Among the others, Kip99p is predicted to have microtubule motor activity and protein homodimerization activity and plays a role in kinesin complex localization. Vps20p is involved in multivesicular body trafficking, and Sec9p is a t-SNARE protein required for secretory vesicle-membrane fusion. The theme that emerged during this analysis points to the upregulation of genes whose products play a role in vesicle transport, localization, and fusion, all of which are required for hyphal tip elongation since cell membrane and wall components are transported via motor proteins to the growing tip to meet the demands of filamentation (76, 77). This could indicate a compensatory mechanism being utilized by C. albicans in an attempt to overcome the potent inhibition of filamentation exerted by compound 9029936 or, alternatively, an attempt to remove the compound from the cell.

GO analysis identified 28 GO terms enriched (overrepresented) in the upregulated gene set in the presence of compound 9029936 under filament-inducing conditions. The most enriched terms included processes such as hyphal growth and filamentation in response to abiotic stimuli (most likely due to the inclusion of genes involved in the negative regulation of filamentation), cellular response to drug, regulation of transcription, and DNA repair (Fig. 4B).

**FIG 5:**
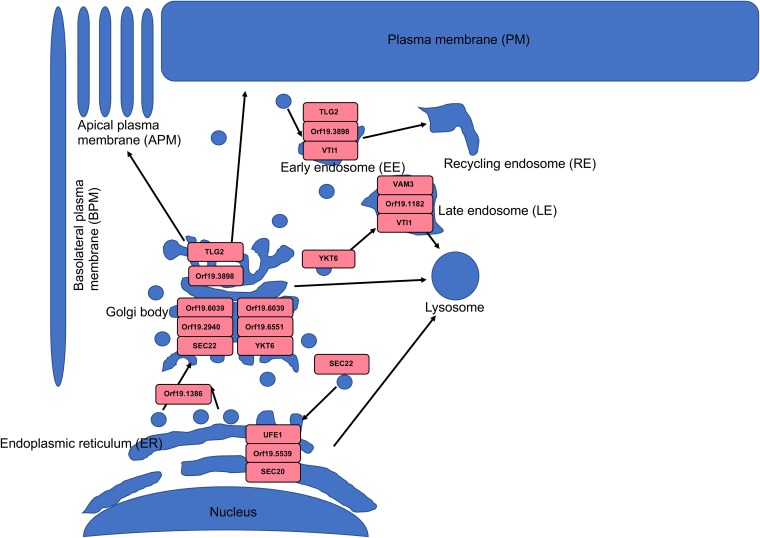
Upregulation of genes in the SNARE interactions in vesicular transport pathway during treatment with compound 9029936. KEGG analysis indicated that a total of 20 genes involved in the SNARE interactions in vesicular transport pathway, indicated in red in the map, were significantly upregulated in C. albicans cells treated with 5 μM compound 9029936 versus untreated control cultures.

As with the downregulated data set, KEGG pathway impact analysis was also performed on the list of genes upregulated by treatment with our leading compound. This analysis detected eight KEGG pathways that were significantly impacted (Table S3). Of these, SNARE interactions in vesicular transport proved to be the pathway most significantly affected. These results support our initial observation based on the heat map of upregulated DEGs (Fig. 4A). Indeed, the morphological transition from yeast to hyphae requires the constant transport and fusion of vesicles into the growing hyphal tip to deliver the components required for the expansion of the membrane as well as the necessary enzymes for cell wall synthesis (76). Furthermore, these results support our previous findings from cytological profiling experiments, in which we observed that the vacuole integrity was compromised by treatment with compound 9029936 (31). A map of SNARE interactions in vesicular transport was generated from the KEGG analysis and is shown in Fig. 5. Genes whose expression is upregulated by treatment with compound 9029936 are shown in red. It is apparent that a large proportion of the components of this pathway show enhanced gene expression in response to this treatment, which again suggests a possible compensatory mechanism used by C. albicans in an attempt to overcome the inhibitory effects of compound 9029936 on filamentation/hypha formation.

10.1128/mSphere.00620-19.4 TABLE S3 Significantly overrepresented KEGG pathways upregulated in C. albicans cells during treatment with compound 9029936. Download Table S3, XLSX file, 0.01 MB. Copyright © 2019 Romo et al. 2019 Romo et al. This content is distributed under the terms of the Creative Commons Attribution 4.0 International license.

In summary, this study provides a comprehensive view of transcriptomic changes associated with the treatment of C. albicans with a novel small-molecule inhibitor of filamentation. Major changes in the expression of key genes and pathways associated with C. albicans pathogenesis processes, including filamentation, iron and phosphate acquisition, metabolic processes, and vesicular transport, among others, provide further information and insight into the antivirulence mode of action of compound 9029936.

## MATERIALS AND METHODS

### Strains, media, and culture conditions.

The wild-type C. albicans strain SC5314 was utilized for these studies. Cell stocks were stored at –80°C, propagated by streaking onto yeast extract-peptone-dextrose (YPD) agar plates (1% [wt/vol] yeast extract, 2% [wt/vol] Bacto peptone, 2% [wt/vol] dextrose, and 1.5% agar), and incubated overnight at 30°C. From these, a loopful of cells was inoculated into flasks (150 ml) containing 25 ml of YPD liquid medium in an orbital shaker at 180 rpm and grown overnight for 14 to 16 h at 30°C. Under these conditions, C. albicans grows as a budding yeast.

### Drugs.

Milligram quantities of the lead small-molecule compound 9029936 were obtained from hit resupply stocks available at Chembridge Corporation (San Diego, CA). Concentrated stock solutions were prepared in dimethyl sulfoxide (DMSO) and stored at −20°C. Working dilutions of the compound at the appropriate final concentration were prepared fresh before each experiment.

### RNA isolation, purification, and sequencing.

The C. albicans SC5314 strain was grown overnight as described above, washed with phosphate-buffered saline (PBS), used to inoculate YPD containing 10% fetal bovine serum (FBS) at a 1:30 dilution, and incubated at 37°C for 90 min in the presence or absence of compound 9029936 at 5 μM as previously described (30). RNA was extracted by using a hot-acid-phenol protocol (78). Three biological replicates were obtained for each condition (treated and untreated). To determine the final RNA concentration and quality, samples were analyzed using a 2100 series bioanalyzer (Agilent Technologies, CA).

RNA sequencing was performed at the Genome Sequencing Facility at the Greehey Children’s Cancer Research Institute at the University of Texas Health Science Center at San Antonio. Briefly, cDNA libraries for RNA-seq analysis were prepared from total RNA samples using an Illumina TruSeq stranded mRNA-seq kit. RNA sequencing was performed using an Illumina HiSeq 2000 machine (San Diego, CA) to obtain 100-bp paired-end reads. After the sequencing run, demultiplexing with CASAVA was employed to generate a fastq file for each sample.

### Transcriptomic analysis.

For analyses of data, the RNA sequencing reads were processed using CLC Genomics Workbench 10.0 (Qiagen). Quality trimming and adapter trimming were performed using default parameters. Reads were mapped to the C. albicans strain SC5314 reference genome (assembly 21) (http://www.candidagenome.org/). Only the uniquely mapped reads were used as the raw expression value, followed by the trimmed mean of M-values (TMM) normalization (79) to eliminate RNA composition bias. Differential gene expression profiling was carried out using the edgeR package implemented in R (79). The Benjamini and Hochberg false-discovery rate (FDR) procedure was used for multiple-hypothesis testing correction (80). Genes with FDR-adjusted *P* value (<0.05) and expression fold changes of more than 2 or less than −2 were considered to be differentially expressed. To validate the RNA sequencing data, we performed quantitative reverse transcription-PCR (qRT-PCR) to measure changes in the amount of mRNA of selected genes between treated and untreated samples (Fig. S1).

10.1128/mSphere.00620-19.1 FIG S1 Confirmatory qRT-PCR analysis of hypha-specific genes differentially expressed during treatment with compound 9029936. Levels of expression of *PHO84*, *ALS3*, and *HWP1* were measured by qRT-PCR in order to confirm results from RNA-seq experiments. The primers used were TTTGTTGGGTTTGTTCGTCA (forward) and GCAATAATGGCACCGACTTT (reverse) for *PHO84*, CAACTTGGGTTATTGAAACAAAAACA (forward) and AGAAACAGAAACCCAAGAACAACCT (reverse) for *ALS3*, and TCAGCCTGATGACAATCCTC (forward) and GCTGGAGTTGTTGGCTTTTC (reverse) for *HWP1*. **, *P* < 0.0015; ***, *P* < 0.0008; ****, *P* < 0.0001. Download FIG S1, PDF file, 0.2 MB. Copyright © 2019 Romo et al. 2019 Romo et al. This content is distributed under the terms of the Creative Commons Attribution 4.0 International license.

Functional enrichment analysis was performed on the differentially expressed genes to identify overrepresented Gene Ontology (GO) biological processes using the Panther classification system (81). Kyoto Encyclopedia of Genes and Genomes (KEGG) (82) pathway enrichment analysis was performed using KOBAS 3.0 software (83). The Benjamini and Hochberg procedure was used for multiple-testing correction for both GO and KEGG pathway analyses, with the cutoff criterion of an FDR of <0.05 (80). Protein-protein association data for Candida albicans (taxon identifier [ID] 5476) were extracted from the STRING (Search Tool for the Retrieval of Interacting Genes) database (84). STRING uses Bayesian models to integrate various sources of data, including genomic context (sequence similarity, genome organization, chromosome synteny, and phylogenetic reconstruction), gene coexpression, biochemical and genetic experimental data, pathway analysis, computational predictions, and literature text mining, to infer protein-protein associations. From a functional perspective, “association” can mean direct physical binding, but it can also mean indirect interaction, such as participation in the same cellular process. A confidence score (S) ranging from 0 to 1 was assigned to each predicted association. Cytoscape 3.7.1 (85) and StringApp (86) were used for the interactome network visualization. The relative expression levels of genes in C. albicans cells treated with 5 μM compound 9029936 versus untreated control cultures are represented in Fig. 3 by a gradient of colors from green (downregulation) to red (upregulation) for log2 fold change.

### Data availability.

The RNA sequencing data were deposited into the GEO database under accession number GSE136116.
