# Supplementary material for: Global Transcriptomic Analysis of the Candida albicans Response to Treatment with a Novel Inhibitor of Filamentation
Source: mSphere. 2019 Sep 11;4(5):e00620-19. doi: 10.1128/mSphere.00620-19 (PMC6739497; doi:10.1128/mSphere.00620-19)
Supplement: TABLE S1 [file mSphere.00620-19-st001.pdf]

Supplementary Table S1. Sequenced reads obtained from the sample set from RNA sequencing experiments.

Paired reads												
	Treated rep 1		Treated rep 2		Treated rep 3		Control rep 1		Control rep 2		Control rep 3	
	Number of sequences	% of total	Number of sequences	% of total	Number of sequences	% of total	Number of sequences	% of total	Number of sequences	% of total	Number of sequences	% of total
Unique reads mapped in pairs	36641248	90.96	34694588	90.06	34208830	89.72	31710950	91.18	34099796	91.77	36043446	90.85
Reads mapped	38764952	96.23	36896654	95.78	36886772	96.75	33534907	96.43	35897847	96.61	38545644	97.16
Total reads after “trim”	40283710	100	38523364	100	38127258	100	34777084	100	37158658	100	39674006	100
