# Supplementary material for: Global Transcriptomic Analysis of the Candida albicans Response to Treatment with a Novel Inhibitor of Filamentation
Source: mSphere. 2019 Sep 11;4(5):e00620-19. doi: 10.1128/mSphere.00620-19 (PMC6739497; doi:10.1128/mSphere.00620-19)

Supplementary Figure S1. Confirmatory qRT-PCR analysis of hyphal specific genes differentially expressed during treatment with compound 9029936 . Levels of expression of *PHO84, ALS3* and *HWP1* were measured by qRT-PCR in order to confirm results from RNA-Sequencing experiments. The primers used were TTTGTTGGGTTTGTTCGTCA (forward) and GCAATAATGGCACCGACTTT (reverse) for *PHO84*, CAACTTGGGTTATTGAAACAAAAACA (forward) and AGAAACAGAAACCCAAGAACAACCT (reverse) for *ALS3*, and TCAGCCTGATGACAATCCTC (forward) and GCTGGAGTTGTTGGCTTTTC (reverse) for *HWP1*. **P < 0.0015; ***P < 0.0008; ****P < 0.0001.

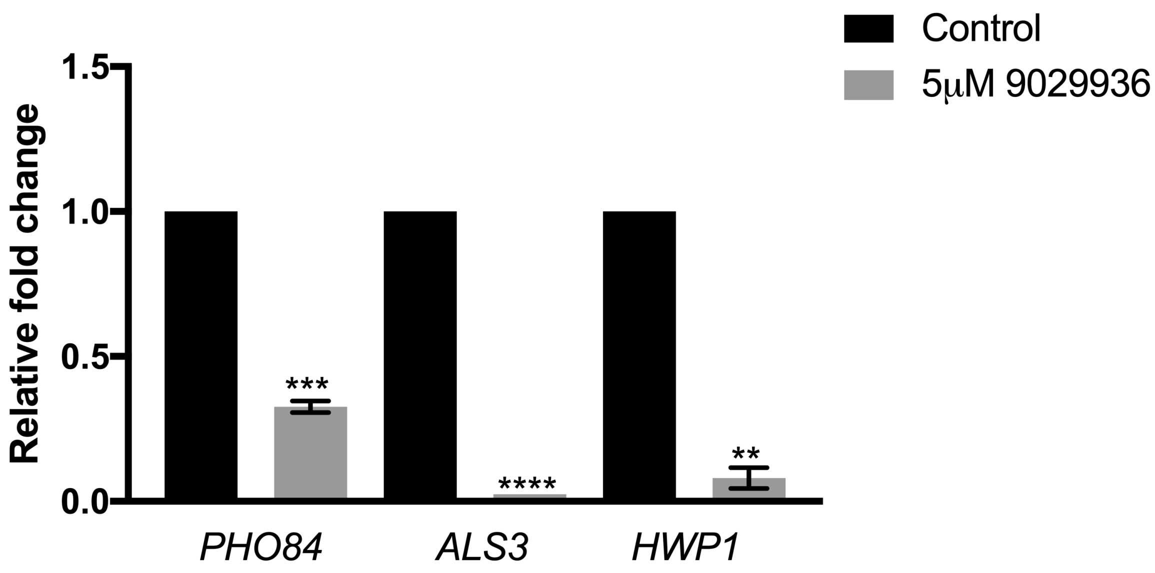

Supplement: FIG S1 [file mSphere.00620-19-sf001.pdf]
